# Impact of co-payment level increase of antidiabetic medications on glycaemic control: an interrupted time-series study among Finnish patients with type 2 diabetes

**DOI:** 10.1186/s12913-020-05952-6

**Published:** 2020-11-27

**Authors:** Piia Lavikainen, Emma Aarnio, Kari Jalkanen, Hilkka Tirkkonen, Päivi Rautiainen, Tiina Laatikainen, Janne Martikainen

**Affiliations:** 1 Faculty of Health Sciences, School of Pharmacy, University of Eastern Finland, P.O.Box. 1627, FI-70211 Kuopio, Finland; 2 Joint Municipal Authority for North Karelia Social and Health Services (Siun Sote), Joensuu, Finland; 3 Department of Public Health Solutions, National Institute for Health and Welfare, Helsinki, Finland; 4 Institute of Public Health and Clinical Nutrition, University of Eastern Finland, Kuopio, Finland

**Keywords:** Type 2 diabetes, Co-payment increase, Glycaemic control

## Abstract

**Background:**

A new special reimbursement scheme (SRS) for non-insulin medications used for treatment of hyperglycaemia in type 2 diabetes (T2D) was implemented in Finland on January 1, 2017. The new SRS affected all community-dwelling Finnish T2D patients as all community-dwelling residents are eligible for reimbursement for prescription medications. The aim of the study was to evaluate the impact of this co-payment increase on glycaemic control among Finnish T2D patients.

**Methods:**

Data on glycaemic control were collected with HbA1c measures from electronic health records from primary health care and specialized care in the North Karelia region, Finland, from patients with a confirmed T2D diagnosis in 2012 who were alive on January 1, 2017 (*n* = 8436). Average HbA1c levels were measured monthly 36 months before and 33 months after the policy change. Consumption of diabetes medications was measured with defined daily doses (DDDs) based on reimbursed medication purchases. Interrupted time series design analysed with segmented regression model was applied to examine the effect of the policy change on average HbA1c levels.

**Results:**

Eight thousand one hundred forty-three T2D patients had at least one HbA1c measurement within 01/2014–9/2019. Mean age of the patients was 68.1 (SD 11.3) years and 53.0% were women. Average time since T2D diagnosis was 11.5 (SD 6.1) years. An estimated increase of 0.81 (95% confidence interval, CI, 0.04–1.58) mmol/mol in average HbA1c levels was detected at the time of the policy change. In subgroup analyses, strongest effects were detected among patients who used only other diabetes medications than insulin or metformin in 2016 (3.56 mmol/mol, 95% CI 2.50–4.62). Meanwhile, yearly consumption of diabetes medications decreased slightly from 618.9 (SD 487.8) DDDs/patient in 2016 to 602.9 (SD 475.6) DDDs/patient in 2017 (*p* = 0.048).

**Conclusions:**

Simultaneously with the increase of the co-payment level, the average HbA1c level increased among T2D patients from the North Karelia region, Finland. This may be explained by the decreased consumption of diabetes medications between 2016 and 2017. Special attention should be allocated to glycaemic control of patients utilizing only other antidiabetic medications than metformin or insulin.

**Supplementary Information:**

The online version contains supplementary material available at 10.1186/s12913-020-05952-6.

## Background

Type 2 diabetes (T2D) is a widespread disease with rapidly increasing prevalence. In Finland alone, the current prevalence of T2D is around 8% and the disease is causing around 15% of the total expenditures of the Finnish health care system [1, 2]. When compared with other Nordic countries, consumption of diabetes medications is high in Finland [3] causing high pharmaceutical expenditure. For this, a new special reimbursement scheme (SRS) for non-insulin medications used for treatment of hyperglycaemia in T2D was implemented in Finland on January 1, 2017 [4–6]. The new SRS affected all community-dwelling Finnish T2D patients utilizing any other diabetes medication than insulin because in Finland, all community-dwelling residents are eligible for reimbursement for prescription medications. During the first year of the new legislation coming into effect, the medication reimbursement costs lowered by 24% (EUR 26 million), but simultaneously the co-payments of T2D patients more than tripled (from EUR 11.9 million to EUR 39.7 million, + 334%) [7].

In previous studies, co-payment increase related to diabetes medications is reported to be associated with decreased use of [8, 9] and lower adherence to medications [10–16], and therefore, with reduced glycaemic control [15, 16]. These changes may have clinical implications in terms of glycaemic control, which in turn is known to be associated with long-term micro- and macrovascular complications [17–19]. Therefore, the aim of the present study was to evaluate the impact of the co-payment increase on glycaemic control among Finnish T2D patients. To provide reasons for possible changes in glycaemic control, we also investigated changes in consumption of diabetes medications before and after the policy change.

## Methods

### Setting

The Finnish SRS and the policy change are described in detail in Lavikainen et al. (2020) and Suviranta et al. (2019) [20, 21]. Briefly, all Finnish residents are eligible for reimbursement for prescription medications purchased from community pharmacies according to three categories based on medical grounds [22] after an initial deductible of EUR 50 per calendar year (since 2015) [23]. The reimbursement is received directly at the community pharmacies. In 2014, 35% of the medication cost in the Basic Refund Category, 65% of the cost in the Lower and 100% of the cost after a fixed co-payment in the Higher Special Refund Categories were reimbursed by the National Health Insurance Scheme [24]. If a patient’s co-payments exceed a certain limit during a calendar year (varying between EUR 572.00 and EUR 612.62 during 2014–2019), the patient becomes eligible for an Additional Refund [7, 23–26]. After reaching the Additional Refund limit, the patient pays only a fixed co-payment for each purchased, reimbursable medication.

Patients having a certain chronic disease, such as diabetes, and meeting medical criteria defined by the Social Insurance Institution of Finland (SII) can be entitled to a special reimbursement for medication costs. To be granted this entitlement for antidiabetic medications other than insulin by the SII, the patient must have a confirmed diabetes diagnosis based on criteria stated by the SII [20]. To receive special reimbursement for GLP-1 (glucagon-like peptide-1) analogues, in addition to confirmed diabetes, other antidiabetic medications need to be first tried and the patient must have a body mass index ≥30 kg/m^2^.

The new SRS implemented on January 1, 2017 lowered the reimbursement level of non-insulin antidiabetic medications from the Higher (100%) to the Lower Special Refund Category (65%) to achieve savings in medication costs [4–6]. This meant a co-payment of 35% of the cost of each purchased non-insulin medication for T2D patients instead of a fixed co-payment (EUR 3.00 in 2014–2015, EUR 4.50 in 2016) for each purchased medication.

### Study design

The regional electronic health records (EHRs) from the Joint Municipal Authority for North Karelia Social and Health Services (Siun sote) were utilized in the present study. These regional EHRs cover both primary health care and specialized care. Extracted data consisted of patients with a confirmed T2D diagnosis (based on 10th revision of International Classification of Diseases, ICD-10 [27], code E11) at the end of 2012 (*n* = 10,204) who were alive on Jan 1, 2017 (*n* = 8436). Data contained information on diagnoses as well as on laboratory assays. EHR data were compiled with information on reimbursed diabetes medication purchases (such as dispensing date and Anatomical Therapeutic Chemical (ATC) classification code [28]) for the years 1995–2010 and 2016–2017 from the Finnish Prescription Register maintained by the SII. In addition, data on entitlements to higher medication reimbursement due to diabetes before 2011 were retrieved from the Special Reimbursement Register maintained also by the SII.

### Outcome measures

In Finland, care of T2D is based on Current Care Guidelines [2] and the general aim of T2D care is to give means for early screening, to prevent complications of diabetes, ensure a balanced treatment and a good life quality for diabetes patients. Typically, HbA1c is used as a measure of long-term blood sugar level reflecting average glycaemic balance over the last 2–8 weeks with values less than 53 mmol/mol (7.0%) considered indicating good treatment balance [2, 29] with some exceptions for older and the most comorbid patients. According to treatment guidelines, HbA1c level should be measured regularly (every 6–12 months).

In the study data, glycaemic control was measured with glycated haemoglobin (HbA1c) with the turbidimetric inhibition immunoanalysis method (TINIA) by the Eastern Finland laboratory (ISLAB, https://www.islab.fi) which is an accredited laboratory and participates external quality surveys. Values were standardised to International Federation of Clinical Chemistry (IFCC) units. Mean HbA1c (mmol/mol) levels were calculated for each month 36 months before and 33 months after the policy change (i.e. January, 2014 – September, 2019) that was introduced on January 1, 2017. In every one-month time-window, data on all patients having measured his/hers HbA1c at that specific window were used. If a patient had more than one HbA1c measurement within a month, the latest one was selected.

Consumption of diabetes medications was estimated with defined daily doses (DDDs, [28]) based on the Prescription register data for the years 2016 and 2017.

### Subgroup analyses

Patient’s age and timing of T2D diagnosis were obtained from the EHRs. Timing of T2D diagnosis was ascertained with the Finnish Prescription Register data on diabetes medication purchases and the Special Reimbursement Register data on entitlements to special refund for diabetes medications maintained by the SII. T2D diagnosis date was considered to be the first occurrence of diabetes medication purchase, entitlement to special refund or T2D diagnosis in the electronic patient database. In subgroup analyses, patients were divided to those with T2D duration from 5 to less than 10 years, 10–15 years, and those with T2D duration > 15 years at the time of policy change.

Patients were classified according to the diabetes medication purchases from the Prescription register data in 2016. The following subgroups were formed: users of metformin (ATC code A10BA02) only, users of metformin and other oral antidiabetic medications (A10BA02 + other A10B), users of only other diabetes medications than insulin or metformin (A10B excluding A10BA02 and A10A), users of insulin and oral antidiabetic medication (including metformin) (A10A + A10B), and users of insulin (A10A) only in 2016.

### Other variables

Information of concordant and discordant diseases was retrieved from the EHRs and they were measured from the time period before the policy change in Dec 31, 2016. Concordant, T2D-coexisting diseases consisted of hypertension (ICD-10: I10), coronary heart disease (I20–I25), atrial fibrillation (I48), heart failure (I50, I11.0, I13.0, I13.2), peripheral arterial diseases (I70.2, I73.9), stroke (incl. SAH, I60, I61, I63, I64, but excluding I63.6), chronic kidney disease (N18, N19), neuropathies (G59, G63, G73, G99), blindness (H54), or diabetes complications (E11.2–E11.8 sublevels). Discordant diseases consisted of cancers (C00–C43, C45–C97), asthma (J45, J46), gout (M10), glaucoma (H40–H42), depression (F32, F33), dementia (F00–F03, G30), mental diseases (F20–F48), chronic obstructive pulmonary disease (J43–J44), rheumatoid and other arthritis (M05–M13, M32, M33, M45), osteoporosis (M80–M85), neuromuscular diseases (G70–G72), or liver diseases excluding cancers (K70–K77).

### Statistical analyses

Differences in baseline characteristics between patients with and without HbA1c measurements during the follow-up were examined using standardized difference that is independent of sample size [30]. Standardized mean difference values > 10% were considered to indicate meaningful differences between the patients.

Interrupted time series [31] design was applied to examine the effect of the policy change on average monthly HbA1c levels. Time periods of 30 days 36 months before and 33 months after the policy change were utilized to define a pre-policy change segment, time of the policy change, and a post- policy change segment. Interrupted time series is a strong quasi-experimental design. It was estimated with segmented linear autoregressive error models [32]. Autocorrelation between the time points was estimated utilizing a Durbin–Watson test [31]. Autocorrelation refers to the dependency of regression residuals over the measured time points. For the results of the Durbin–Watson test, *p* < 0.05 was considered to indicate statistically significant serial autocorrelation. Autocorrelation was automatically adjusted for in the regression models when needed.

In the primary analysis, data on the total population were utilized. However, subgroup analyses were performed based on age and T2D duration at the time of the policy change as well as diabetes medication use in 2016. Sensitivity analyses against the primary analysis were conducted ruling out time-periods of 1–6 months after policy change (January–June, 2017) to examine the impact of potential lag time on the effect of diabetes medications on HbA1c levels. In addition, in the second sensitivity analysis, two-month time periods instead of one-month periods were applied to stabilize the potential variation due to short time-windows and to increase sample sizes within time-windows. All the analyses were conducted with SAS version 9.4 (SAS Institute Inc., Cary, North Carolina, USA).

### Ethics statement

Use of the data was approved by the Ethics Committee of the Northern Savonia Hospital District (diary number 81/2012). The study protocol was also approved by the register administrator, the Joint Municipal Authority for North Karelia Social and Health Services (Siun sote). A separate permission to link data on medication purchases and entitlements to special reimbursements was achieved from the SII (diary number 110/522/2018). Only register-based data were utilized and thus, consent from the patients was not needed.

## Results

Patients with at least one HbA1c measurement during 1/2014–9/2019 (*n* = 8143) were on average 68.1 years (SD 11.3) old at the baseline and 53.0% were female (Table 1). A bit over two thirds (69.2%) were on good glycaemic control (i.e. HbA1c less than 53 mmol/mol) at the time of the policy change. Roughly a third (35.4%) of patients had concordant diseases only in addition to T2D and 9.7% discordant diseases only, whereas 24.6% had both concordant and discordant diseases and 30.3% had neither of them. Most patients used only metformin in 2016, followed by users of insulin and oral antidiabetic medication. Patients without HbA1c measurements (*n* = 293 or 3.5%) during the time period were on average healthier (no concordant or discordant diseases), but, however, more of them died during the follow-up when compared to patients with HbA1c measurements (7.9% vs. 5.8%, respectively, see Additional file 1).

**Table 1:** Characteristics of T2D patients on Jan 1, 2017 as frequencies (proportions) unless otherwise stated

	All patients	Used only metformin in 2016	Used metformin and other OAD in 2016	Used only other diabetes medications than insulin or metformin in 2016	Used insulin and OAD (inc. metformin) in 2016	Used only insulin in 2016
*n* (%)	8143 (100)	2271 (27.9)	1430 (17.6)	726 (8.9)	2112 (25.9)	577 (7.1)
Mean age, years (SD)	68.1 (11.3)	69.5 (10.7)	68.0 (10.8)	71.8 (10.8)	69.4 (10.9)	74.0 (11.9)
< 75 years old	5203 (63.9)	1519 (66.9)	1040 (72.7)	407 (56.1)	1423 (67.4)	281 (48.7)
≥75 years old	2940 (36.1)	752 (33.1)	390 (27.3)	319 (43.9)	689 (32.6)	296 (51.3)
Female	4315 (53.0)	1104 (48.6)	809 (56.6)	361 (49.7)	1244 (58.9)	317 (54.9)
Mean time since T2D diagnosis, yrs. (SD)	11.5 (6.1)	8.9 (4.1)	10.9 (4.4)	10.3 (4.8)	15.4 (6.2)	17.2 (7.9)
Duration of T2D						
≤ 10 years	4243 (52.1)	1659 (73.1)	738 (51.6)	432 (59.5)	466 (22.1)	119 (20.6)
10–15 years	1976 (24.3)	529 (18.9)	456 (31.9)	193 (26.6)	645 (30.5)	132 (22.9)
> 15 years	1924 (23.6)	183 (5.1)	236 (16.5)	101 (13.9)	1001 (47.4)	326 (56.5)
Concordant diseases only	2880 (35.4)	722 (31.8)	511 (35.7)	276 (38.0)	861 (40.8)	215 (37.3)
Discordant diseases only	787 (9.7)	235 (10.4)	151 (10.6)	59 (8.1)	159 (7.5)	56 (9.7)
Both concordant and discordant diseases	2005 (24.6)	412 (18.1)	291 (20.4)	169 (23.3)	630 (29.8)	212 (37.3)
Without concordant or discordant diseases	2471 (30.3)	902 (39.7)	477 (33.4)	222 (30.6)	462 (21.9)	94 (16.3)
Good glycaemic control (< 53 mmol/mol) in 2016	5636 (69.2)	2133 (93.9)	964 (67.4)	608 (83.8)	709 (33.6)	224 (38.8)
Died during 2017	391 (4.8)	62 (2.7)	36 (2.5)	53 (7.3)	102 (4.8)	70 (12.1)
Died during the follow-up	474 (5.8)	77 (3.4)	44 (3.1)	58 (8.0)	123 (5.8)	84 (14.6)

*Abbreviations*: *OAD* oral antidiabetic drug, *T2D* type 2 diabetes

^†^Concordant diseases: hypertension, coronary heart disease, atrial fibrillation, heart failure, peripheral arterial diseases, stroke (incl. SAH), chronic kidney disease, neuropathies, blindness, or diabetes complications (E11.2-E11.8 sublevels)

^‡^Discordant diseases: cancers, asthma, gout, glaucoma, depression, dementia, mental diseases, rheumatoid and other arthritis, osteoporosis, neuromuscular diseases, or liver diseases excl. cancers

Note: 1027 patients did not use any diabetes medication in 2016

**Fig. 1:**
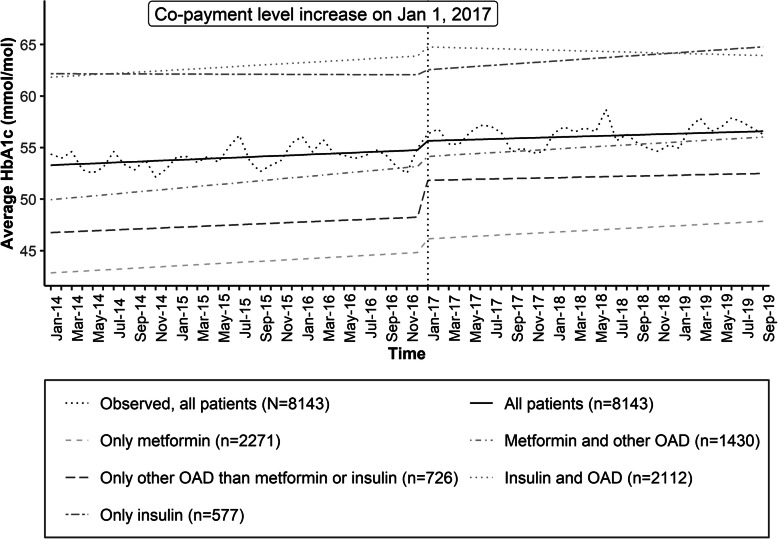
Observed time series of average glycaemic control for all patients by month. Estimated trend lines show predicted values from the segmented regression analysis for all patients and by drug groupings

In the primary analysis, for the total population, the average HbA1c level was 52.4 (95% confidence interval, CI, 51.9–52.9) mmol/mol at the baseline on January, 2014 and HbA1c level increased by 0.07 mmol/mol (95% CI 0.04–0.09) per month until December 2016 (Fig. 1, Table 2). An estimated average increase of 0.81 (95% CI 0.04–1.58) mmol/mol was detected in HbA1c levels at the time of policy change on January, 2017. Thereafter, the average HbA1c level remained stable until September 2019.

**Table 2:** Parameter estimates (95% confidence intervals) for average HbA1c (mmol/mol) levels over the follow-up

	Baseline level at 01/2014	Pre-policy change trend in 01/2014–12/2016	Change in level at the time of the policy change in 01/2017	Post-policy change trend in 02/2017–9/2019
All patients (*n* = 8143)	52.40 (51.88 to 52.91)	0.07 (0.04 to 0.09)	0.81 (0.04 to 1.58)	0.03 (− 0.03 to 0.08)
Subgroup analyses:				
Patients aged < 75 years (*n* = 5203)	52.05 (51.50 to 52.60)	0.06 (0.04 to 0.09)	0.79 (− 0.04 to 1.62)	0.04 (− 0.02 to 0.10)
Patients aged ≥75 years (*n* = 2940)	53.39 (52.59 to 54.19)	0.06 (0.02 to 0.10)	1.53 (0.40 to 2.66)	0.00 (−0.08 to 0.09)
≤ 10 years duration of T2D (*n* = 4243)	47.35 (46.80 to 47.90)	0.06 (0.04 to 0.09)	0.68 (−0.03 to 1.39)	0.05 (− 0.01 to 0.11)
> 10–15 years duration of T2D (*n* = 1976)	53.15 (52.41 to 53.89)	0.09 (0.06 to 0.13)	0.85 (−0.29 to 1.99)	0.02 (− 0.06 to 0.09)
> 15 years duration of T2D (*n* = 1924)	59.76 (58.57 to 60.95)	0.03 (−0.02 to 0.09)	1.49 (− 0.11 to 3.09)	0.02 (− 0.11 to 0.15)
Used only metformin in 2016 (*n* = 2271)	42.79 (42.44 to 43.14)	0.06 (0.04 to 0.07)	1.28 (0.83 to 1.73)	0.05 (0.02 to 0.09)
Used metformin and other OAD in 2016 (*n* = 1430)	49.85 (49.17 to 50.53)	0.09 (0.06 to 0.12)	0.92 (−0.10 to 1.94)	0.06 (−0.01 to 0.13)
Used only other diabetes medications than insulin or metformin in 2016 (n = 726)	46.71 (45.99 to 47.43)	0.04 (0.01 to 0.08)	3.56 (2.50 to 4.62)	0.02 (−0.06 to 0.10)
Used insulin and OAD (incl. metformin) in 2016 (*n* = 2112)	61.78 (60.88 to 62.68)	0.06 (0.02 to 0.10)	0.92 (−0.39 to 2.22)	−0.03 (− 0.12 to 0.07)
Used only insulin in 2016 (*n* = 577)	62.18 (60.98 to 63.38)	−0.00 (− 0.06 to 0.05)	0.42 (−1.28 to 2.12)	0.07 (− 0.06 to 0.20)

*Abbreviations*: *OAD* oral antidiabetic drug;, *T2D* type 2 diabetes

In subgroup analyses, stronger point estimates for immediate changes in HbA1c levels at the time of the policy change than in the primary analysis were detected among patients utilizing only other diabetes medications than insulin or metformin in 2016 (3.56 mmol/mol, 95% CI 2.50–4.62) (Table 2). In addition, stronger point estimates for immediate changes in HbA1c levels at the time of the policy change were also detected among patients aged ≥75 years (Table 2).

In sensitivity analyses, ruling out time-periods of 1 to 6 months (January–June, 2017) after the policy change diluted the effect of the policy change to statistically non-significant except in the analysis excluding the period from January to May (Table 3). Neither using two-month instead of one-month time-windows nor restricting the population to those who survived until October 2019 altered the results from the primary analysis (Table 3).

**Table 3:** Parameter estimates (95% confidence intervals) from sensitivity analyses for average HbA1c (mmol/mol) levels over the follow-up

	Baseline level at 01/2015	Pre-policy change trend in 01/2014–12/2016	Change in level at the time of the policy change in 01/2017	Post-policy change trend in 02/2017–9/2019
Primary result	52.40 (51.88 to 52.91)	0.07 (0.04 to 0.09)	0.81 (0.04 to 1.58)	0.03 (− 0.03 to 0.08)
Results from sensitivity analyses when...				
...excluding 01/2017	52.52 (51.50 to 53.54)	0.06 (0.02 to 0.10)	1.00 (−0.01 to 2.01)	0.03 (−0.07 to 0.13)
...excluding 01–02/2017	52.44 (51.89 to 53.00)	0.06 (0.04 to 0.09)	0.72 (−0.13 to 1.57)	0.04 (−0.02 to 0.10)
...excluding 01–03/2017	52.81 (51.79 to 53.23)	0.06 (0.03 to 0.10)	0.76 (−0.31 to 1.83)	0.04 (−0.04 to 0.12)
...excluding 01–04/2017	52.55 (51.82 to 53.28)	0.06 (0.02 to 0.09)	0.99 (−0.10 to 2.07)	0.04 (−0.05 to 0.12)
...excluding 01–05/2017	52.59 (51.53 to 53.66)	0.05 (0.01 to 0.10)	1.28 (0.14 to 2.41)	0.03 (−0.08 to 0.15)
...excluding 01–06/2017	52.76 (51.73 to 53.80)	0.04 (0.00 to 0.09)	1.37 (−0.05 to 2.79)	0.04 (−0.09 to 0.16)
...applying 2-month time windows	53.04 (52.64 to 53.44)	0.09 (0.05 to 0.12)	0.86 (0.28 to 1.44)	0.06 (−0.01 to 0.12)
...restricting the population to those who survived until Oct 1, 2019 (*n* = 7669)	51.95 (51.46 to 52.45)	0.07 (0.05 to 0.10)	1.00 (0.25 to 1.75)	0.03 (−0.02 to 0.09)

In total, consumption of diabetes medications decreased from 11,619 daily DDDs in 2016 to 11,446 daily DDDs in 2017 among those who were alive on Jan 1, 2018 (Table 4). Meanwhile, the number of purchases increased from 52,050 purchases in 2016 to 56,764 purchases in 2017 and the number of users decreased from 6793 patients in 2016 to 6780 patients in 2017. Number of purchases increased from an average 7.7 (SD 7.4) purchases per patient in 2016 to an average 8.4 (SD 8.2) purchases per patient in 2017 (*p* < 0.001). Consumption of diabetes medications per patient per purchase decreased from 81.5 (SD 60.9) DDDs/patient/purchase in 2016 to 73.6 (SD 59.3) DDDs/patient/purchase in 2017 (*p* < 0.001). Even the total consumption of medications calculated as DDDs decreased, the total yearly consumption of diabetes medications per patient who had purchases did not change between 2016 and 2017. In total, consumption of SGLT2 (sodium/glucose cotransporter 2) inhibitors increased heavily from 144,018 DDDs in 2016 to 245,228 DDDs in 2017 whereas consumption of other diabetes medications decreased during the same period (Additional file 2).

**Table 4:** Consumption of diabetes medications in 2016 and 2017.

	***N*** of users (***N*** of purchases)		Total consumption, DDDs		Purchases/patient (SD)			DDDs/patient/purchase (SD)			DDDs/patient/year (SD)		
	2016	2017	2016	2017	2016	2017	***P*** value	2016	2017	***P*** value	2016	2017	***P*** value
All patients (*n* = 8143)	7116 (55,104)	7036 (58,212)	12,065	11,621	7.7 (7.5)	8.3 (8.2)	< 0.001	79.9 (61.3)	72.9 (59.4)	< 0.001	618.9 (487.8)	602.9 (475.6)	0.048
Patients alive on Jan 1, 2018 (*n* = 8025)	6793 (52,050)	6780 (56,764)	11,619	11,446	7.7 (7.4)	8.4 (8.2)	< 0.001	81.5 (60.9)	73.6 (59.3)	< 0.001	624.3 (489.0)	616.2 (477.2)	0.326
*Subgroup analyses:*													
Used only metformin in 2016 (*n* = 2271)	2271 (9632)	2184 (10,036)	1678	1681	4.2 (4.2)	4.6 (4.6)	0.007	63.6 (42.6)	61.1 (42.2)	< 0.001	269.6 (129.0)	280.9 (145.4)	0.006
Used metformin and other OAD in 2016 (*n* = 1430)	1430 (12,946)	1412 (14,009)	2806	2700	9.1 (7.7)	9.9 (8.7)	0.005	79.1 (44.0)	70.3 (44.9)	< 0.001	716.3 (268.6)	697.9 (299.2)	0.084
Used only other diabetes medications than insulin or metformin in 2016 (*n* = 726)	726 (4009)	694 (4316)	714	701	5.5 (5.3)	6.2 (5.6)	0.016	65.0 (41.9)	59.3 (39.7)	< 0.001	358.8 (169.4)	368.6 (193.9)	0.309
Used insulin and OAD (incl. metformin) in 2016 (*n* = 2112)	2112 (25,656)	2088 (26,503)	6046	5745	12.1 (9.0)	12.7 (9.8)	0.061	86.0 (72.3)	79.2 (70.7)	< 0.001	1044.9 (572.1)	1004.9 (559.7)	0.022
Used only insulin in 2016 (*n* = 577)	577 (2861)	548 (2893)	821	737	5.0 (2.5)	5.3 (3.2)	0.064	104.8 (77.0)	93.0 (70.4)	< 0.001	519.6 (440.6)	490.8 (413.1)	0.259
Patients alive on Oct 1, 2019 (*n* = 7669)	6730 (51,486)	6721 (56,235)	11,526	11,372	7.7 (7.3)	8.4 (8.2)	< 0.001	81.7 (60.7)	73.8 (59.3)	< 0.001	625.1 (488.6)	617.6 (477.9)	0.366

*Abbreviations*: *DDD* defined daily dose, *OAD* oral antidiabetic drug

Of those utilizing only other diabetes medications than insulin or metformin in 2016 and surviving until Jan 1, 2018 (*n* = 726), 6.8% started using metformin and 5.9% insulin in addition to other diabetes medications than insulin or metformin in 2017 (Table 5).

**Table 5:** Changes in medication use between 2016 and 2017 by medication subgroups among those who survived until Jan 1, 2018 (*n* = 8025)

	Used only metformin in 2017	Used metformin and other OAD in 2017	Used only other diabetes medications than insulin or metformin in 2017	Used insulin and OAD (incl. metformin) in 2017	Used only insulin in 2017	Did not use antidiabetic medications in 2017
Used only metformin in 2016 (*n* = 2271)	1967 (89.0)	147 (6.7)	10 (0.5)	16 (0.7)	1 (0.1)	68 (3.1)
Used metformin and other OAD in 2016 (*n* = 1430)	42 (3.0)	1164 (83.5)	76 (5.5)	101 (7.3)	3 (0.2)	8 (0.6)
Used only other diabetes medications than insulin or metformin in 2016 (*n* = 726)	2 (0.3)	46 (6.8)	563 (83.7)	40 (5.9)	0	22 (3.3)
Used insulin and OAD (incl. metformin) in 2016 (*n* = 2112)	11 (0.6)	23 (1.1)	17 (0.9)	1889 (94.0)	63 (3.1)	7 (0.4)
Used only insulin in 2016 (*n* = 577)	0	0	1 (0.2)	35 (6.9)	461 (90.9)	10 (2.0)

*Abbreviations*: *OAD* oral antidiabetic drug

## Discussion

A small 0.8 mmol/mol (~ 0.08%) immediate increase in average HbA1c levels during the first month after the new SRS was detected in glycaemic control among T2D patients from the North Karelia region, Finland. However, the largest immediate increase (3.56 mmol/mol, 95% CI 2.50–4.62, or 0.33%) was observed among patients utilizing only other diabetes medications than insulin or metformin in 2016. Patients were observed to purchase smaller packages and more frequently after the policy change than before it.

The estimated yearly increase of 0.84 mmol/mol (12*0.07 mmol/mol, 0.08% units) in average HbA1c levels observed in this study until the policy change at the beginning of 2017 is a bit over the magnitude of the estimated increase of 0.75 mmol/mol (0.07% units in HbA1c) per year that was observed in our previous study among the same population in 2011–2016 [33]. The small differences in results are due to differences in densities of follow-up (monthly level in the current study vs. yearly level in the paper by Nazu et al. (2019)) in addition to differences in lengths of follow-up [33]. In the current study, an additional, immediate increase of 0.81 mmol/mol (0.08%) was detected at the time of the policy change in the current study. Thereafter, HbA1c values continued to increase 0.36 mmol/mol (0.03%) per year. Compared with other international publications on changes in treatment balance over time, rates of 1.4–1.5 mmol/mol (0.12–0.14% units in HbA1c) of increase per year are reported [34, 35]. The lower rate of increase in our studies may reflect the early detection and active treatment of T2D patients in the North Karelia region [36]. In addition, SGLT2 inhibitors became reimbursable in 2016 and the price of newer medications, such as GLP-1 analogues, has decreased over the study period increasing their use and, thus, reducing the use of insulins.

The new SRS has already been reported to effect patient’s medication use, cause financial difficulties to purchase diabetes medications, and worsen patient’s satisfaction to diabetes care [20, 21]. In our study, almost 13% of the patients utilizing only other medications than insulin or metformin in 2016 started using metformin or insulin in addition to other diabetes medications in 2017. In our previous study, 28% of patients with T2D reported they discontinued non-insulin diabetes medication use due to financial reasons and 8% had initiated insulin use due to the same reasons within the first 11 months after the implementation of the new SRS [20]. In another study, we also observed that almost half (47%) of the study participants reported some kind of an effect of the co-payment level increase on their life in an open-ended question 11 months after the new SRS coming into effect [21]. Most commonly reported effects were economic effects (33%), such as increased expenditure (17%) or difficulty in purchasing medicines (9%), after the co-payment level increased. However, only 2% reported they had discontinued diabetes medication use.

The previously reported effects of the new SRS [20, 21] may show as a decline in treatment balance observed in the current study. Increases in co-payment levels are also reported to decrease adherence to diabetes medications [15, 16, 37] which may further show as a decline in treatment balance [15]. In addition, the observed results of elevated HbA1c levels at the time of the policy change may be explained by the decreased consumption of diabetes medications between 2016 and 2017. We observed that the number of purchases increased while the number of users decreased due to mortality indicating that the patients purchased smaller packages and more frequently after the policy change than before it. Similar findings were observed at the national level: the consumption of diabetes medications decreased by 1% between August, 2016 and August, 2017 although the number of users increased by 3% [38]. However, the decreasing trend in consumption of diabetes medications observed in both studies may be explained by stockpiling at the end of 2016; patients anticipated the upcoming higher prices and utilized the benefits of possibly reaching the Additional Refund limit. In another previous national level study, the annual co-payment increase was estimated to be EUR 157 on average among those utilizing DPP-4 (dipeptidyl peptidase 4) inhibitors or GLP-1 analogues, while corresponding figure for patients using older antidiabetic medicines (e.g., metformin and sulfonylureas) was EUR 12 [39]. This may be the reason why the largest immediate effects of the new SRS were seen among patients utilizing only other diabetes medications than insulin or metformin in 2016 in this study. To remind, insulins remained in the Higher Special Refund Category (100%) at the time of the policy change. Furthermore, increasing prices may delay initiation of diabetes medications other than insulin or metformin. Further examination on use of these medications is needed among new T2D patients with a longer follow-up.

Strengths of our study are inclusion of all patients with a diagnosed T2D in 2012 in the North Karelia region and application of data on all available HbA1c measurements, and, thus, avoiding selection bias. In addition, all the municipalities of the North Karelia use the same regional laboratory and the same standardized methods for HbA1c testing. Utilization of register-based data allows us to avoid recall bias, too.

However, our study also includes some weaknesses. At the same time of the introduction of the new SRS, the Joint Municipal Authority for North Karelia Social and Health Services (Siun sote) was launched in the North Karelia region, Eastern Finland. Siun sote is a consortium of municipalities and organizes health care services for 14 municipalities in North Karelia instead of each municipality arranging its own services. However, how introduction of new structure for organizing health care services could affect T2D patients and their glycaemic control, remains unknown. To our knowledge, treatment routines remained similar as before the introduction of the consortium. Still, we were not able to separate the effect of the new SRS of that of the new service structure. It should be noted that all the patients included in our study were diagnosed with T2D at least 1 year before the start of the follow-up of HbA1c development and at least 4 years before the policy change in Jan, 2017. In addition, according to our previous study, younger patients with T2D were poorly monitored compared with the older patients in the North Karelia region [33]. Furthermore, we did not have information on patients using private health care services. However, as persons utilizing private health care services are not likely to use only private health care services in the care of T2D due to, for example, economic issues, this is not a big concern.

## Conclusions

T2D is a lifelong, progressing disease that, among others, affects quality of life, introduces comorbidities and increases mortality risk [2]. Higher HbA1c levels (> 53 mmol/mol or 7.0%) in turn are reported to increase the risk of micro- and macrovascular complications [17–19]. We observed that the co-payment level increase of antidiabetic medications had the strongest, immediate average effect on glycaemic control among those who were utilizing only other diabetes medications than insulin or metformin at the time of the policy change. The observed HbA1c increase of 3.56 mmol/mol (0.33%) at the time of the policy change equals over 17% increase in the risk of microvascular complications, almost 5% increase in the risk of myocardial infarctions and 9% increase in the risk of diabetes related deaths according to the risk models from the UKPDS study [40]. Therefore, future studies are warranted to monitor long-term incidence of complications as well as related health and economic outcomes as an outcome of this policy change in this specific subgroup of patients.

## Supplementary Information

**Additional file 1.** Characteristics of T2D patients at the time of the co-payment increase on Jan 1, 2017 as frequencies (proportions) unless otherwise stated.

**Additional file 2.** Number of purchases and total DDDs by ATC subgroups in 2016 and 2017.

## Data Availability

Due to individual privacy law, data sharing is not possible and are not publicly available. An anonymised version of the data is available upon reasonable request from the corresponding author with appropriate permissions of the Joint Municipal Authority For North Karelia Social and Health Services (Siun sote) and the Social Insurance Institute.

## Abbreviations

ATC: Anatomical Therapeutic Chemical
CI: Confidence interval
DDD: Defined daily dose
DPP-4: Inhibitors of dipeptidyl peptidase 4
EHRs: Electronic health records
GLP-1: Glucagon-like peptide-1
ICD-10: 10th revision of International Classification of Diseases
SGLT2: Sodium/glucose cotransporter 2
SII: The Social Insurance Institute of Finland
SRS: Special reimbursement scheme
T2D: Type 2 diabetes
