# Supplementary material for: Impact of co-payment level increase of antidiabetic medications on glycaemic control: an interrupted time-series study among Finnish patients with type 2 diabetes
Source: BMC Health Serv Res. 2020 Nov 27;20:1095. doi: 10.1186/s12913-020-05952-6 (PMC7694920; doi:10.1186/s12913-020-05952-6)
Supplement: Supplementary file 1 — Additional file 1. Characteristics of T2D patients at the time of the co-payment increase on Jan 1, 2017 as frequencies (proportions) unless otherwise stated. [file 12913_2020_5952_MOESM1_ESM.pdf]

Additional file 1. Characteristics of type 2 diabetes patients at the time of the co-payment increase on Jan 1, 2017 as frequencies (proportions) unless otherwise stated.

	T2D patients alive on Jan 1, 2017 (n=8436)	HbA1c measurements during the follow-up (n=8143)	No HbA1c measurement during the follow-up (n=293)	SMD (%)
Mean age, yrs (SD)	69.9 (11.5)	68.1 (11.3)	64.8 (15.2)	39.5
<75 years old	5424 (64.3)	5203 (63.9)	221 (75.4)	25.3
≥75 years old	3012 (35.7)	2940 (36.1)	72 (24.6)	25.3
Female	3958 (46.9)	3828 (47.0)	130 (44.4)	5.3
Mean time since T2D diagnosis, yrs (SD)	11.4 (6.1)	11.5 (6.1)	8.9 (5.0)	45.6
Duration of T2D				
<10 years	4441 (52.6)	4243 (52.1)	198 (67.6)	32.0
10-15 years	2037 (24.2)	1976 (24.3)	61 (20.8)	8.3
>15 years	1958 (23.2)	1924 (23.6)	34 (11.6)	32.0
Good glycaemic control (<53 mmol/mol) in 2016	5929 (70.3)	5636 (69.2)	NA	
T2D + concordant diseases only	2913 (34.5)	2880 (35.4)	33 (11.3)	59.5
T2D + discordant diseases only	812 (9.6)	787 (9.7)	25 (8.5)	3.9
T2D + both concordant and discordant diseases	2048 (24.3)	2005 (24.6)	43 (14.7)	25.2
T2D without concordant or discordant diseases	2663 (31.6)	2471 (30.3)	192 (65.5)	75.2
Died during the follow-up	497 (5.9)	474 (5.8)	23 (7.9)	8.3

Abbreviations: SMD, standardized mean difference; T2D, type 2 diabetes.

[†]Concordant diseases: hypertension, coronary heart disease, atrial fibrillation, heart failure, peripheral arterial diseases, stroke (incl. SAH), chronic kidney disease, neuropathies, blindness, or diabetes complications (E11.2-E11.8 sublevels).

[‡]Discordant diseases: cancers, asthma, gout, glaucoma, depression, dementia, mental diseases, rheumatoid and other arthritis, osteoporosis, neuromuscular diseases, or liver diseases excl. cancers.
