# Supplementary material for: Impact of co-payment level increase of antidiabetic medications on glycaemic control: an interrupted time-series study among Finnish patients with type 2 diabetes
Source: BMC Health Serv Res. 2020 Nov 27;20:1095. doi: 10.1186/s12913-020-05952-6 (PMC7694920; doi:10.1186/s12913-020-05952-6)
Supplement: Supplementary file 2 — Additional file 2. Number of purchases and total DDDs by ATC subgroups in 2016 and 2017. [file 12913_2020_5952_MOESM2_ESM.pdf]

**Additional file 2**. Number of purchases and total DDDs by ATC subgroups in 2016 and 2017 among type 2 diabetes patients who survived until Jan 1, 2018 (n=8025).

| | **2016** | | **2017** | |
|---|---|---|---|---|
| | **Purchases** | **DDD** | **Purchases** | **DDD** |
| Insulin (A10A) | 11,300 | 1,347,694 | 12,184 | 1,342,181 |
| Metformin (A10BA02) | 21,866 | 1,592,232 | 22,164 | 1,499,515 |
| Sulfonylureas (A10BB) | 729 | 67,225 | 541 | 50,347 |
| Combinations of oral blood glucose lowering drugs (A10BD) | 2451 | 195,500 | 2722 | 192,115 |
| Glitazones (A10BG) | 358 | 24,541 | 255 | 19,177 |
| DPP-4 inhibitors (A10BH, gliptins) | 11,484 | 740,239 | 12,689 | 706,970 |
| GLP-1 analogues (A10BJ) | 1414 | 121,361 | 1669 | 114,285 |
| SGLT2 inhibitors (A10BK) | 2299 | 144,018 | 4407 | 245,228 |
| Glinides (A10BX02) | 149 | 8221 | 133 | 7864 |

Abbreviations: ATC, Anatomical Therapeutic Chemical; DDD, defined daily dose.
